# Factors associated with exacerbations among adults with asthma according to electronic health record data

**DOI:** 10.1186/s40733-019-0048-y

**Published:** 2019-01-18

**Authors:** Rebecca E. Greenblatt, Edward J. Zhao, Sarah E. Henrickson, Andrea J. Apter, Rebecca A. Hubbard, Blanca E. Himes

**Affiliations:** 1 Department of Biostatistics, Epidemiology and Informatics, Perelman School of Medicine, University of Pennsylvania, Philadelphia, PA 19104 USA; 2 Division of Allergy-Immunology, Children’s Hospital of Philadelphia, Philadelphia, PA 19104 USA; 3 Institute for Immunology, University of Pennsylvania, Philadelphia, PA 19104 USA; 4 Pulmonary, Allergy and Critical Care Division, Perelman School of Medicine, University of Pennsylvania, Philadelphia, PA 19104 USA

**Keywords:** Chronic obstructive pulmonary disease, Chronic bronchitis, Emphysema, Obesity, Sinusitis

## Abstract

**Background:**

Asthma is a chronic inflammatory lung disease that affects 18.7 million U.S. adults. Electronic health records (EHRs) are a unique source of information that can be leveraged to understand factors associated with asthma in real-life populations. In this study, we identify demographic factors and comorbidities associated with asthma exacerbations among adults according to EHR-derived data and compare these findings to those of epidemiological studies.

**Methods:**

We obtained University of Pennsylvania Hospital System EHR-derived data for asthma encounters occurring between 2011 and 2014. Regression analyses were performed to model asthma exacerbation frequency as explained by age, sex, race/ethnicity, health insurance type, smoking status, body mass index (BMI) and various comorbidities. We analyzed data from the National Health and Nutrition Examination Survey (NHANES) from 2001 to 2012 to compare findings with those from the EHR-derived data.

**Results:**

Based on data from 9068 adult patients with asthma, 33.37% had at least one exacerbation over the four-year study period. In a proportional odds logistic regression predicting number of exacerbations during the study period (levels: 0, 1–2, 3–4, 5+ exacerbations), after controlling for age, race/ethnicity, sex, health insurance type, and smoking status, the highest odds ratios (ORs) of significantly associated factors were: *chronic bronchitis* (2.70), *sinusitis* (1.50), *emphysema* (1.39), *fluid and electrolyte disorders* (1.35), *class 3 obesity* (1.32), and *diabetes* (1.28). An analysis of NHANES data showed associations for *class 3 obesity*, *anemia* and *chronic bronchitis* with exacerbation frequency in an adjusted model controlling for age, race/ethnicity, sex, financial class and smoking status.

**Conclusions:**

EHR-derived data is helpful to understand exacerbations in real-life asthma patients, facilitating design of detailed studies and interventions tailored for specific populations.

**Electronic supplementary material:**

The online version of this article (10.1186/s40733-019-0048-y) contains supplementary material, which is available to authorized users.

## Background

Asthma is an inflammatory lung disease characterized by periods of airflow obstruction that affects over 18.7 million American adults [1, 2], and whose total yearly costs in the U.S. are over $81.9 billion [3]. Prevalence of asthma is higher in women than men, and in black vs. white persons [4–6]. Additionally, asthma mortality is higher for adults than children, 30% higher for women than men, and 75% higher for black than white persons [2]. Clinical therapy following established guidelines successfully controls asthma symptoms in most patients [7]. However, episodes of worsening symptoms termed *exacerbations* remain a considerable source of asthma morbidity, mortality and healthcare costs [8–11].

Many observational and prospective studies have identified sociodemographic, clinical and environmental factors that are associated with asthma exacerbations among adults. Previous studies, including The Severe Asthma Research Program (SARP)-3, one of the largest characterization studies of severe asthma consisting of 75% adult subjects, found that exacerbation frequency was associated with blood eosinophils, body-mass index (BMI), bronchodilator responsiveness, and comorbidities, including sinusitis and gastro-esophageal reflux disease (GERD) [12, 13]. People with asthma who also have chronic obstructive pulmonary disease (COPD) are at increased risk for exacerbations vs. those who only have asthma [14], while people with COPD who also have asthma are at increased risk for exacerbations vs. those who only have COPD [15]. The study of these individuals with both asthma and COPD, now referred to as asthma-COPD overlap (ACO), has been a topic of recent interest [16].

Electronic health record (EHR)-derived data offers convenient and low-cost access to longitudinal data for large numbers of patients that can be leveraged to understand demographic and comorbidity relationships [17–19]. Although data collected via EHRs is subject to bias and missingness that most epidemiological studies and clinical trials are able to control for, EHR-derived data has the benefit of capturing a larger amount of information corresponding to real-life, diverse patient populations [20, 21]. EHR-derived data has been used successfully to identify subjects for asthma genomics studies, and its potential to study exacerbations and comorbidity patterns among asthma patients has been demonstrated [22–25]. Here, we used EHR-derived data from 9068 adults with asthma who utilized the University of Pennsylvania Hospital System (UPHS) to identify demographic factors and comorbid conditions associated with increased exacerbation frequency. We compare these results to those obtained by analyzing data from the National Health and Nutrition Examination Survey (NHANES), a Center for Disease Control & Prevention (CDC)-led cross-sectional study, as well as those obtained from a previously published study conducted with data from (SARP)-3 [13, 26].

## Methods

A detailed description of methods, including variable ascertainment and analysis of NHANES data, is provided in the Additional file.

### Study population

De-identified EHR-derived data corresponding to UPHS patients was obtained from Penn Data Store (PDS), a clinical data warehouse that supports medical research and patient care initiatives [27, 28]. Specifically, patient-level data for adult (i.e., aged 18 years or older) encounters occurring January 1, 2011 to December 31, 2014 that contained at least one asthma International Classification of Disease, Ninth Revision (ICD-9) diagnosis code (i.e., 493*) were obtained [29]. Variables extracted included sex, age, race/ethnicity, health insurance type, smoking history, encounter type (i.e., outpatient, inpatient, or emergency), height, weight, and all ICD-9 codes recorded for each patient. BMI was classified into 5 categories: *not overweight or obese* (< 25.0 kg/m^2^), *overweight* (25.0 to < 30.0 kg/m^2^), *class 1 obese* (30.0 to < 35.0 kg/m^2^), *class 2 obese* (35.0 to < 40.0 kg/m^2^) and *class 3 obese* (≥ 40.0 kg/m^2^). Corticosteroid and respiratory agent medication history for each patient was captured from codified entries in the EHR as well as Natural Language Processing (NLP)-extracted values from encounter notes. The University of Pennsylvania Institutional Review Board approved our study (protocol number 824789). The final study population consisted of 9,068 patients who had complete BMI, health insurance type, and smoking history data. Inclusion criteria and description of variable ascertainment are included in the Additional file.

*Asthma exacerbation* was defined as an encounter with (1) a primary ICD-9 code for asthma and (2) an oral corticosteroid (OCS) order. Because a large number of patients had COPD-related comorbidity codes and thus, their exacerbations could be coded as asthma or COPD, a second outcome termed *chronic airway obstruction exacerbation* was defined as having primary ICD-9 codes for chronic and acute bronchitis (490*, 491*), emphysema (492*), asthma (493*), chronic airway obstruction not otherwise specified (496*), shortness of breath (786.05) or wheezing (786.07), while still requiring the encounter to include an oral corticosteroid order.

### Characteristics of patients with insufficient preventative care

To infer whether some persons with exacerbations might have less controlled disease, we compared patients with *inpatient* or *emergency* exacerbation visits who did and did not have at least one *outpatient* visit in the preceding 6 months. Chi-squared tests were performed to determine significance.

### Characteristics of patients with ACO

To assess whether patients with asthma only vs. ACO differ in their demographic, comorbidity and exacerbation frequency characteristics, patients with a diagnosis of *emphysema* and/or *chronic bronchitis*, termed *ACO patients*, were compared to asthma patients without a diagnosis of *emphysema* or *chronic bronchitis.* Chi-squared tests were performed to determine significance.

### Statistical analysis

Statistical analyses were conducted in R [30]. We performed proportional odds logistic regression models using the R *MASS* package to obtain crude and adjusted odds ratios (ORs) [31]. The outcome variable consisted of four ordered categories according to the number of exacerbations a subject had during the study period: *0*, *1–2*, *3–4*, and *5+ exacerbations.* Demographic and comorbidity variables were included in models as independent predictors. Description of comorbidity variable selection can be found in the Additional file. Parallel slopes tests were performed to determine whether the assumption made by the proportional odds logistic regression that a given predictor increases the probability of moving from one outcome level to the next identically for each step was appropriate [see Additional file 1: Table E4].

## Results

### EHR-based study population characteristics

Comparison of 9068 study subjects vs. those excluded due to missing BMI, health insurance type, and/or smoking history data found statistically significant differences in distribution of age, sex, race, health insurance type*, chronic bronchitis, sinusitis, obstructive sleep apnea, pulmonary circulation disorders* and *diabetes* (*p* < .05) [see Additional file 1 Table E2]. Compared to all UPHS patients encountered during the study period, our study subjects were more likely to be *female* (74.7% vs. 59.5%), and *black or African American* (51.5% vs. 27.9%), suggesting that trends in local asthma disparities by sex and race/ethnicity mirror known U.S. disparities [4–6, 32, 33]. Among study subjects, 6042 (66.6%) had no asthma exacerbations during the study period, 2639 (29.1%) had 1–2, 273 (3.0%) had 3–4, and 114 (1.3%) had 5+. Encounters with a primary ICD-9 code of asthma, regardless of oral steroid order, were most likely to be *outpatient* (77.3%) compared to *emergency* (17.4%) or *inpatient* (5.3%). 97.1% of *inpatient* asthma encounters had an associated oral steroid order compared to 20.9% of *outpatient* asthma encounters and 18.8% of *emergency* asthma encounters.

The distribution of demographic and comorbidity variables across exacerbation levels is shown in Table 1. Prevalence of each comorbidity increased directionally with exacerbation count. According to asthma-related medication data extracted from EHRs [Table 2], 6920 (77.8%) of study subjects had at least one prescription for a controller therapy (i.e., inhaled corticosteroid (ICS) or ICS/long acting beta agonist (LABA) combination drug). Other drugs, including anticholinergics, anti-IgE monoclonal antibody, leukotriene receptor antagonists (LTRAs) and SABA/anticholinergic combinations, were present at a higher rate as the number of exacerbations increased.

**Table 1:** Patient Characteristics by Exacerbation Count Levels. Comorbidity categories included in the adjusted model are included. For each category, N (%) for raw data are shown

	Number of Exacerbations			
	0	1–2	3–4	5+
	(*N* = 6042)	(*N* = 2639)	(*N* = 273)	(*N* = 114)
Age (years)				
18–30	1534 (25.39)	370 (14.02)	23 (8.42)	12 (10.53)
31–40	1141 (18.88)	448 (16.98)	44 (16.12)	18 (15.79)
41–50	1207 (19.98)	569 (21.56)	70 (25.64)	41 (35.96)
51–60	1095 (18.12)	666 (25.24)	74 (27.11)	23 (20.18)
61–70	753 (12.46)	386 (14.63)	45 (16.48)	16 (14.04)
71–80	312 (5.16)	200 (7.58)	17 (6.23)	4 (3.51)
Race				
White	3034 (50.22)	1252 (47.44)	99 (36.26)	12 (10.53)
Black or African American	3008 (49.78)	1387 (52.56)	174 (63.74)	102 (89.47)
Sex				
Male	1576 (26.08)	623 (23.61)	68 (24.91)	28 (24.56)
Female	4466 (73.92)	2016 (76.39)	205 (75.09)	86 (75.44)
BMI (kg/m^2^)				
Not overweight or obese (< 25)	1479 (24.48)	537 (20.35)	54 (19.78)	18 (15.79)
Overweight (25 to < 30)	1715 (28.38)	685 (25.96)	66 (24.18)	21 (18.42)
Class 1 obese (30 to < 35)	1286 (21.28)	584 (22.13)	63 (23.08)	29 (25.44)
Class 2 obese (35 to < 40)	780 (12.91)	352 (13.34)	50 (18.32)	21 (18.42)
Class 3 obese (≥ 40)	782 (12.94)	481 (18.23)	40 (14.65)	25 (21.93)
Health Insurance Type				
Private insurance	3498 (57.89)	1409 (53.39)	116 (42.49)	22 (19.30)
Medicaid	1348 (22.31)	544 (20.61)	89 (32.60)	51 (44.74)
Medicare	1196 (19.79)	686 (25.99)	68 (24.91)	41 (35.96)
Smoking History				
Never	3499 (57.91)	1356 (51.38)	116 (42.49)	48 (42.11)
Quit	1716 (28.40)	843 (31.94)	112 (41.03)	43 (37.72)
Yes	827 (13.69)	440 (16.67)	45 (16.48)	23 (20.18)
Chronic Bronchitis				
No	5711 (94.52)	2215 (83.93)	192 (70.33)	71 (62.28)
Yes	331 (5.48)	424 (16.07)	81 (29.67)	43 (37.72)
Emphysema				
No	5965 (98.73)	2553 (96.74)	256 (93.77)	104 (91.23)
Yes	77 (1.27)	86 (3.26)	17 (6.23)	10 (8.77)
Sinusitis				
No	4066 (67.30)	1548 (58.66)	156 (57.14)	62 (54.39)
Yes	1976 (32.70)	1091 (41.34)	117 (42.86)	52 (45.61)
Pulmonary Circulation Disorders				
No	5749 (95.15)	2403 (91.06)	239 (87.55)	89 (78.07)
Yes	293 (4.85)	236 (8.94)	34 (12.45)	25 (21.93)
Fluid and Electrolyte Disorders				
No	5054 (83.65)	1985 (75.22)	175 (64.10)	53 (46.49)
Yes	988 (16.35)	654 (24.78)	98 (35.90)	61 (53.51)
Obstructive Sleep Apnea				
No	5233 (86.61)	2110 (79.95)	202 (73.99)	68 (59.65)
Yes	809 (13.39)	529 (20.05)	71 (26.01)	46 (40.35)
Diabetes (uncomplicated)				
No	5168 (85.53)	2023 (76.66)	187 (68.50)	58 (50.88)
Yes	874 (14.47)	616 (23.34)	86 (31.50)	56 (49.12)

**Table 2:** Patient Medication Classes by Exacerbation Count Levels. Patients were assigned to oral corticosteroid and respiratory agent medication classes if they had at least one order or prescription for a medication corresponding to each class in 2011–2014 UPHS EHRs. For each category, N (%) for raw data are shown

Medication Class	Number of Exacerbations				*P*-Value
	0	1–2	3–4	5+	
	(*N* = 6042)	(*N* = 2639)	(*N* = 273)	(*N* = 114)	
Anticholinergic	525 (8.69)	862 (32.66)	154 (56.41)	95 (83.33)	< 1e-15
Anti-IgE	20 (0.33)	29 (1.1)	12 (4.4)	10 (8.77)	<1e-15
Epinephrine	209 (3.46)	120 (4.55)	20 (7.33)	16 (14.04)	7.73e-9
ICS	2583 (42.75)	1307 (49.53)	170 (62.27)	72 (63.16)	< 1e-15
ICS/LABA	2811 (46.52)	1682 (63.74)	235 (86.08)	109 (95.61)	< 1e-15
LABA	80 (1.32)	65 (2.46)	15 (5.49)	10 (8.77)	8.58e-14
LTRA	1594 (26.38)	1013 (38.39)	177 (64.84)	101 (88.6)	< 1e-15
OCS	2604 (43.1)	2639 (100)	273 (100)	114 (100)	< 1e-15
PDE-4 inhibitor	3 (0.05)	14 (0.53)	2 (0.73)	2 (1.75)	1.51e-8
SABA	5688 (94.14)	2589 (98.11)	272 (99.63)	114 (100)	< 1e-15
SABA/anticholinergic	295 (4.88)	377 (14.29)	80 (29.3)	56 (49.12)	< 1e-15
Terbutaline	31 (0.51)	25 (0.95)	11 (4.03)	11 (9.65)	< 1e-15
Xanthine	60 (0.99)	81 (3.07)	25 (9.16)	17 (14.91)	< 1e-15

*ICS* inhaled corticosteroid, *LABA* long-acting β_2_-agonist, *LTRA* leukotriene receptor antagonist, *OCS* oral corticosteroid, *PDE-4* phosphodiesterase-4, *SABA* short-acting β_2_-agonist

### Characteristics of patients with insufficient preventative care

Of 166 patients who had at least one *inpatien*t exacerbation visit without an *outpatient* visit in the preceding 6 months, 80.72% were *female*, 83.73% were *black or African American*, 43.98% were on *Medicaid* insurance, and 32.53% were on *Medicare* insurance*.* Of 460 patients who had at least one *emergency* exacerbation visit without an *outpatient* visit in the preceding 6 months, 78.04% were *female*, 92.61% were *black or African American*, 45.22% were on *Medicaid* insurance, and 20.65% were on *Medicare* insurance.

### Characteristics of patients with ACO

*Chronic bronchitis* and *emphysema* were comorbidities in 9.7 and 2.0% of subjects, respectively. Asthma only vs. ACO patients were significantly different in all demographic and comorbidity categories except sex and *sinusitis* (*p* < 0.05) [Table 3]. Most notably, ACO patients were more likely to have asthma exacerbations; 577 (60.7%) of 951 ACO patients had at least one asthma exacerbation, compared to 2449 (30.2%) of 8117 asthma only patients. ACO patients were also more likely to be older and *black or African American*, and have a positive smoking history and diagnosis corresponding to comorbidities other than *sinusitis* (*p* < 0.05).

**Table 3:** Overall characteristics of patients with ACO vs. asthma only. For each category, N (%) for raw data are shown

		ACO	Asthma Only	*P*-Value
		*N* = 951	*N* = 8117	
Exacerbation Count				< 1e-15
	0	374 (39.33)	5668 (69.83)	
	1–2	446 (46.90)	2193 (27.02)	
	3–4	84 (8.83)	189 (2.33)	
	5+	47 (4.94)	67 (0.83)	
Age (years)				< 1e-15
	18–30	19 (2.00)	1920 (23.65)	
	31–40	40 (4.21)	1611 (19.85)	
	41–50	195 (20.50)	1692 (20.85)	
	51–60	337 (35.44)	1521 (18.74)	
	61–70	236 (24.82)	964 (11.88)	
	71–80	124 (13.04)	409 (5.04)	
Race				< 1e-15
	White	311 (32.70)	4086 (50.34)	
	Black or African American	640 (67.30)	4031 (49.66)	
Sex				
	Male	251 (26.39)	2044 (25.18)	
	Female	700 (73.61)	6073 (74.82)	
BMI (kg/m^2^)				0.014
	Not overweight or obese (< 25)	213 (22.40)	1875 (23.10)	
	Overweight (25 to < 30)	224 (23.55)	2263 (27.88)	
	Class 1 obese (30 to < 35)	218 (22.92)	1744 (21.49)	
	Class 2 obese (35 to < 40)	137 (14.41)	1066 (13.13)	
	Class 3 obese (≥ 40)	159 (16.72)	1169 (14.40)	
Health Insurance Type				< 1e-15
	Private insurance	244 (25.66)	4801 (59.15)	
	Medicaid	280 (29.44)	1752 (21.58)	
	Medicare	427 (44.90)	1564 (19.27)	
Smoking History				< 1e-15
	Never	210 (22.08)	4809 (59.25)	
	Quit	486 (51.10)	2228 (27.45)	
	Yes	255 (26.81)	1080 (13.31)	
Chronic Bronchitis				–
	No	72 (7.57)	8117 (100)	
	Yes	879 (92.43)	0 (0)	
Emphysema				–
	No	761 (80.02)	8117 (100)	
	Yes	190 (19.98)	0 (0)	
Sinusitis				0.22
	No	594 (62.46)	5238 (64.53)	
	Yes	357 (37.54)	2879 (35.47)	
Pulmonary Circulation Disorders				< 1e-15
	No	752 (79.07)	7728 (95.21)	
	Yes	199 (20.93)	389 (4.79)	
Fluid and Electrolyte Disorders				< 1e-15
	No	492 (51.74)	6775 (83.47)	
	Yes	459 (48.26)	1342 (16.53)	
Obstructive Sleep Apnea				< 1e-15
	No	645 (67.82)	6968 (85.84)	
	Yes	306 (32.18)	1149 (14.16)	
Diabetes (uncomplicated)				< 1e-15
	No	554 (58.25)	6882 (84.79)	
	Yes	397 (41.75)	1235 (15.21)	

### Factors associated with asthma exacerbations

**Fig. 1:**
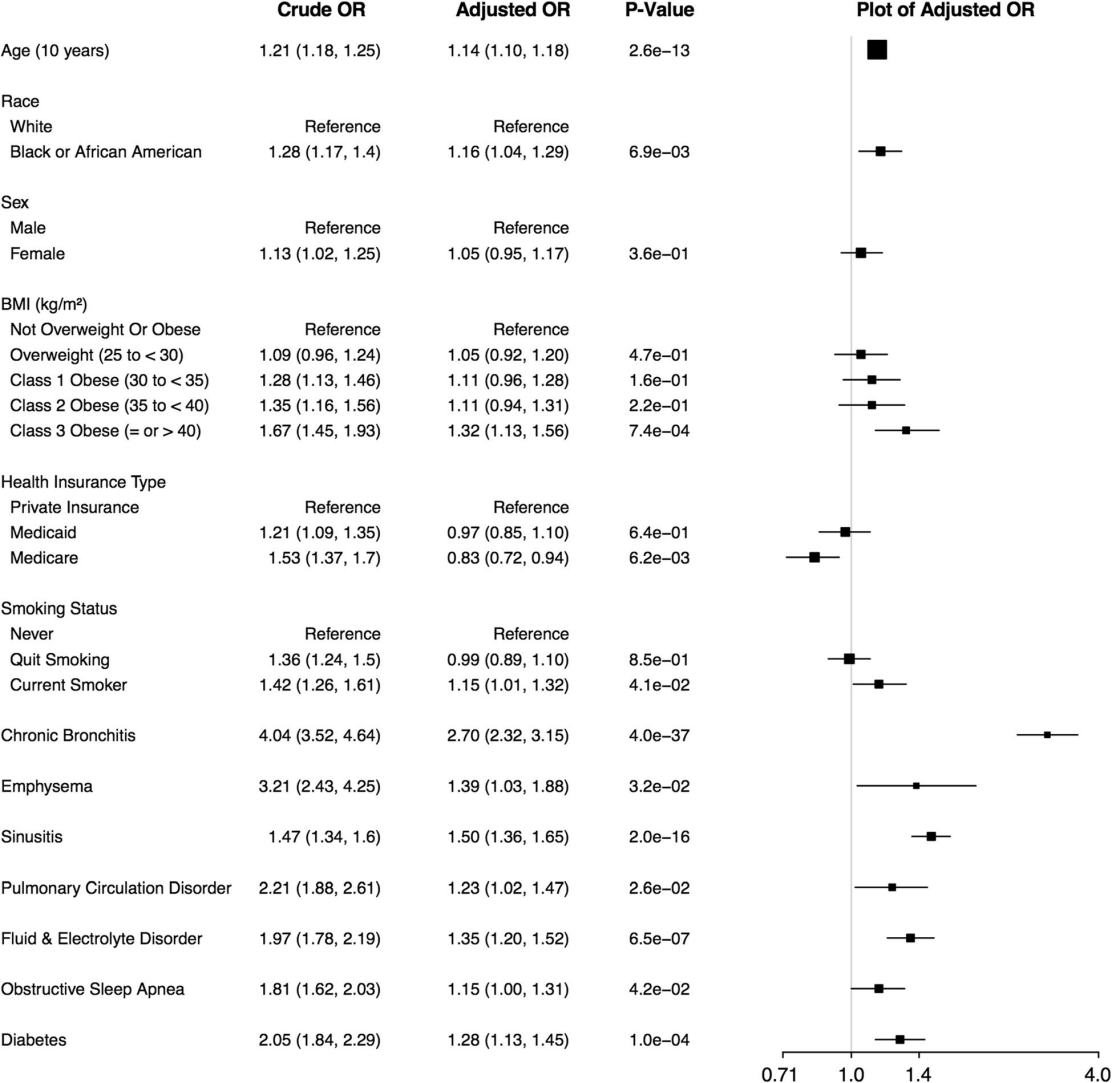
Factors Associated with Exacerbation Levels. Crude and adjusted odds ratios (ORs) for exacerbation levels (0, 1–2, 3–4, 5+ exacerbations) as the outcome were obtained using unadjusted and adjusted proportional odds logistic regression models. Crude and adjusted ORs and 95% confidence intervals (CIs) are shown and adjusted values are plotted

According to unadjusted analyses, each demographic and comorbidity variable was significantly associated with being in an increased exacerbation frequency category (*p* < 0.05) [Fig. 1]. In the adjusted model that included all variables listed in Fig. 1, race *black or African American* vs. *white* remained significant but with lower effect (adjusted odds ratio (adj. OR): 1.16). Of the BMI levels, only *class 3 obese* vs. *not overweight or obese* remained a significant predictor (adj. OR: 1.32). Health insurance type *Medicare* vs. *Private insurance* had an opposite effect compared to that in the unadjusted model, becoming negatively associated with increased exacerbation frequency category (adj. OR: 0.83), while *Medicaid* insurance became not-significant. Being a *current smoker* compared to a *never smoker* remained significantly associated with exacerbation frequency in the adjusted model (adj. OR: 1.15), while *quit smoking* did not. All comorbid conditions were significant in the adjusted model, with *chronic bronchitis* having the greatest effect: 2.70 times increased odds of being in a higher exacerbation frequency category. The strongest violations of the parallel slopes assumption were for race and health insurance type, with the odds ratios associated with race *black or African American, Medicaid* and *Medicare* increasing as the exacerbation threshold increased [see in Additional file 1 Table E4].

### Factors associated with chronic airway obstruction exacerbations

With the broader exacerbation definition, the number of patients with at least one exacerbation increased from 3026 (33.4%) to 3625 (40.0%) and the number of patients with 5+ exacerbations increased from 114 (1.3%) to 328 (3.6%) [see Additional file 1: Tables E5 & E6]. Crude and adjusted proportional odds logistic regression models to predict the broader *chronic airway obstruction exacerbation* outcome produced results similar to those of the *asthma exacerbation* outcome for sex, BMI, health insurance type, smoking history, and *diabetes*. An expected increase in the association of *chronic bronchitis* and *emphysema* with exacerbations was observed. Notable differences from the adjusted model included that race and *obstructive sleep apnea* were no longer significant predictors; *sinusitis*, *pulmonary circulation disorders* and *fluid and electrolyte disorders* remained significant and had increased adjusted ORs and *class 2 obesity* also became significant [see Additional file 1: Table E7].

### Comparison of EHR-based results to NHANES

**Fig. 2:**
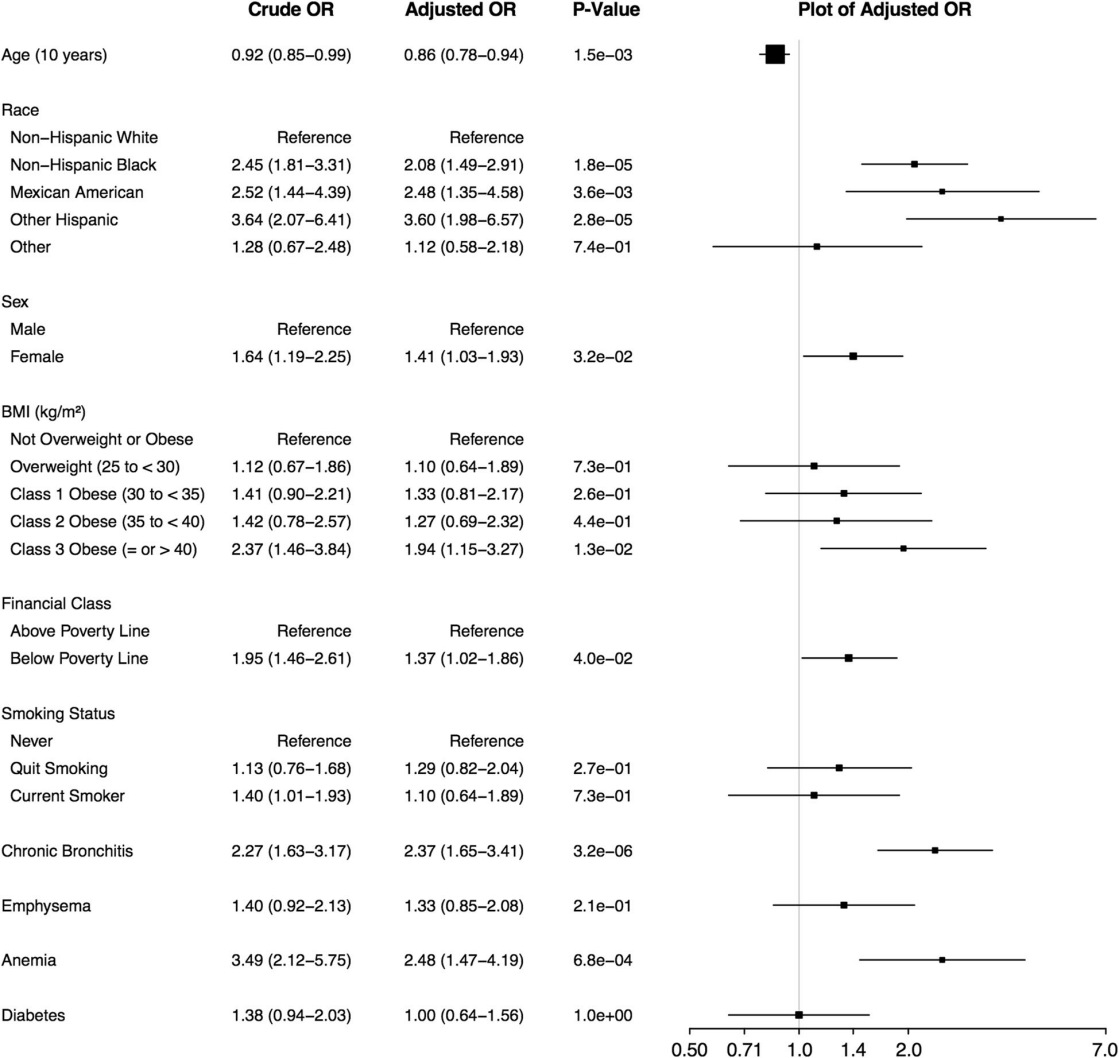
Factors Associated with Exacerbation in NHANES. Crude and adjusted odds ratios (ORs) with > 1 exacerbation as the outcome were obtained using unadjusted and adjusted survey logistic regression models. Crude and adjusted ORs and 95% confidence intervals (CIs) are shown and adjusted values are plotted

Characteristics of 2071 NHANES respondents with asthma and complete data, of which 318 (weighted percentage: 12.73%; raw percentage: 15.35%) had at least one exacerbation, are provided in Additional file 1: Table E8. NHANES subjects had similar ages to those of the EHR-based subjects, but differed in most other categories. Of note, race/ethnicity categories differed (no Hispanic, Mexican American or other subjects included in EHR-based analyses) and NHANES had higher rates of smokers and obese subjects. Some of the comorbidities obtained for the EHR-derived subjects were not available in NHANES and vice-versa, although some overlapped, including *emphysema, chronic bronchitis,* and *diabetes* [see Additional file 1: Table E9]. Medications reported by subjects for the same drug categories as were available for the EHR-based subjects are in Additional file 1: Table E10. Adjusted survey logistic regression model results [Fig. 2] found that odds of exacerbation increased for *non-Hispanic black* race compared to *non-Hispanic white* (adj. OR 2.08), sex *female* compared to *male* (adj. OR 1.41), *class 3 obese* vs. *not overweight or obese* BMI (adj. OR 1.94) and Poverty-to-Income Ratio (PIR) *<* *1* (associated with living at or below the poverty line) (adj. OR: 1.37). Among comorbidities, *anemia* (adj. OR: 2.48) and *chronic bronchitis* (adj. OR: 2.37) were associated with exacerbations.

## Discussion

According to EHR-derived data from 9068 adults with asthma, the factors most strongly associated with asthma exacerbations were the comorbid conditions *chronic bronchitis*, *sinusitis*, *emphysema*, *fluid and electrolyte disorders*, and *class 3 obesity*. Although *black or African American* race*, Medicaid* and *Medicare* health insurance type were positive predictors in unadjusted analyses, their effect decreased, became not-significant, and became opposite, respectively, in adjusted analyses. Similarly, positive smoking history was a predictor of exacerbations in unadjusted analyses, but the effect decreased for *Yes* smoking history and became not-significant for *Quit* smoking history in adjusted analyses. We compared these results to those obtained for NHANES and a previously published SARP study [13]. Despite differences in ascertainment and variables captured across these three studies that limits the comparisons that can made, contrasting their results highlights what is unique about each study and identifies common demographic and comorbidity associations that generalize across diverse study populations.

The definition of asthma in each study population differed: (1) EHR-based subjects were identified on the basis of billing codes and a history of albuterol prescription, (2) NHANES subjects were identified based on self-report, and (3) SARP enrolled subjects based on a physician diagnosis of asthma and, usually, high-dose ICS use and a second controller therapy [13]. Comparison of medication classes of EHR-based subjects over the 4-year study period [Table 2] to those of NHANES subjects, which corresponded to self-reported medication use in the past month [see Additional file 1: Table E10], suggested that EHR-based subjects had more severe chronic airways disease. Because medication frequency across all classes of drugs increased with exacerbation frequency in both EHR-based and NHANES subjects, disease severity and frequency of exacerbations were confounded in these studies. In contrast, SARP analyses were able to explicitly control for disease severity [13]. Ascertaining exacerbations in NHANES was based only on affirmative response to a question about urgent care visits in the prior year, while SARP also included OCS use in its three-level definition of exacerbation frequency, making the latter definition more reflective of severe exacerbations. NHANES had less comorbidity data available than the other two studies. Although a major benefit of a study like SARP is the availability of lab values and pulmonary function tests collected similarly for all subjects, SARP enrolled only nonsmokers without COPD, limiting the applicability of its findings to a smaller group than the EHR-based study or NHANES. A benefit of the EHR-based cohort was its larger sample size (*n* = 9068) than SARP (*n* = 709) and NHANES (*n* = 2071).

According to EHR-based results, *black or African American* race was positively correlated with exacerbation frequency, while sex was not significant. In NHANES, *black or African American* race and *female* sex were both positively correlated. In SARP-3, race and sex were not significant after controlling for variables including some comorbidities, bronchodilator reversibility, blood eosinophil count, and IgE levels [13], although in a replication population (SARP-1 + 2), *female* sex was significant. The finding that *female* sex was not significant in EHR-based subjects was maintained for the broader *chronic airway obstruction exacerbation* outcome, in which *black or African American* race became not-significant [see Additional file 1: Table E7].

BMI, specifically, *class 3 obese* in EHR-based and NHANES subjects, was a significant predictor of asthma exacerbations in all study populations. In terms of comorbidities, EHR-based results found seven categories to be significantly associated with asthma exacerbation frequency [Fig. 1], while in NHANES *anemia* and *chronic bronchitis* were significant, and in SARP, *sinusitis* and *GERD* [13]. The comparison between asthma only and ACO patients and the high odds ratios for *chronic bronchitis* and *emphysema* in EHR-based results are consistent with NHANES and previous studies of people with ACO showing that they had more exacerbations than people with asthma alone [14, 34]. Because SARP excluded subjects with COPD, a comparison cannot be made with that study. *Sinusitis* was a significant factor in both EHR-based subjects and SARP. Because NHANES did not contain information about sinusitis, a comparison cannot be made with that study. Our results are consistent with the existence of known asthma endotypes, such as obese and allergic asthma, but future work with additional clinical variables or information extracted from EHR notes is necessary to better elucidate the relationship between endotypes and comorbidities.

Our analysis of patients with *inpatient* and *emergency* exacerbations that were not preceded by an *outpatient* visit in the prior 6 months, found that patients with less preventative care were more likely to be *black or African American*, and have *Medicaid* or *Medicare* health insurance compared to the overall study population (*p* < 0.001). While these trends may not generalize to other study populations, they could aid in the design of local interventions to reduce asthma exacerbations. Of note, medication adherence data (e.g. fill data) was not available and is likely an important predictor of asthma control.

In addition to bias in how clinical data is collected, missingness related to data capture during encounters, and missingness due to patients using other health providers or not seeking care, there are other limitations of EHR-derived data worth highlighting. First, important variables that are known to influence disease are not adequately captured, including socioeconomic status [34] and biological variables that are measured in epidemiologic studies. Another limitation is the use of billing codes and medication data to assign disease status to subjects. For example, the association of *fluid and electrolyte disorders* could reflect increased number of laboratory tests associated with inpatient visits, rather than potential asthma-related processes (e.g., use of bronchodilators causing hypokalemia [35]). More broadly, our definition of an asthma exacerbation requires a primary visit code for asthma with an oral corticosteroid order, which may be incomplete, especially if medication history was not fully captured. Our classification of COPD may be inaccurate as it relied only on past diagnoses of emphysema or chronic bronchitis, and did not require evidence of fixed airway obstruction. Additionally, as COPD exacerbations are sometimes treated with antibiotics alone without steroids, exacerbation count may have been underestimated in the chronic airway obstruction analysis.

## Conclusion

Our results suggest that comorbid factors *chronic bronchitis*, *sinusitis*, *emphysema*, *fluid and electrolyte disorders*, *class 3 obesity*, and *diabetes* are strongly associated with exacerbations among adults with asthma according to EHR-derived data. *COPD*, *obesity* and *sinusitis* were the most generalizable factors across EHR-based and two epidemiological study populations. In the UPHS EHR population specifically, race and health insurance type were strongly associated with exacerbations among those patients who had 5+ exacerbations, as well as those with less preventive care. Our study demonstrates that EHR-derived data is helpful to understand the characteristics of real-life people with asthma. Future efforts to reduce bias and limitations inherent in EHRs will further improve our ability to identify modifiable risk factors and tailor interventions to decrease asthma exacerbations in diverse populations.

## Additional file

Additional file 1: This file contains a detailed description of the methods along with Figure E1 and Tables E1 through E10. Figure E1 is a correlation matrix of the comorbidity and demographic variables used in the multivariable EHR model. Tables E1 through E7 provide additional information on patient characteristics, variable selection, and sensitivity analyses for the EHR data. Tables E8 through E10 contain patient characteristics, variable selection and medication information for NHANES data. (DOCX 242 kb)

## Abbreviations

ACO: Asthma-COPD Overlap
BMI: Body Mass Index
CDC: Center for Disease Control
COPD: Chronic Obstructive Pulmonary Disease
EHR: Electronic Health Record
GERD: Gastro-esophageal Reflux Disease
ICS: Inhaled Corticosteroid
LABA: Long Acting Beta Agonist
NHANES: National Health and Nutrition Examination Survey
NLP: Natural Language Processing
OCS: Oral Corticosteroid
OR: Odds Ratio
PDS: Penn Data Store
SARP: The Severe Asthma Research Program
