# Supplementary material for: Factors associated with exacerbations among adults with asthma according to electronic health record data
Source: Asthma Res Pract. 2019 Jan 18;5:1. doi: 10.1186/s40733-019-0048-y (PMC6339400; doi:10.1186/s40733-019-0048-y)
Supplement: Supplementary file 1 — This file contains a detailed description of the methods along with Figure E1 and Tables E1 through E10. Figure E1 is a correlation matrix of the comorbidity and demographic variables used in the multivariable EHR model. Tables E1 through E7 provide additional information on patient characteristics, variable selection, and sensitivity analyses for the EHR data. Tables E8 through E10 contain patient characteristics, variable selection and medication information for NHANES data. (DOCX 242 kb) [file 40733_2019_48_MOESM1_ESM.docx]

Factors Associated with Exacerbations Among Adults with Asthma According to Electronic Health Record Data

Rebecca E Greenblatt, Edward J Zhao, Sarah E Henrickson, Andrea J Apter, Rebecca A Hubbard, Blanca E Himes

Additional File 1

**Inclusion criteria and variable ascertainment.**

We restricted our study to patients who (1) were between 18 and 80 years old at the beginning of the study period, (2) were followed for at least 3 years during the study period, as determined by earliest and latest encounter date among all visits, to increase the likelihood that study patients would have utilized UPHS during the study period, and (3) had at least one visit with a primary asthma diagnosis code during the study period and received albuterol at least once between 2004 and 2014 to increase the likelihood that study patients sought asthma care primarily within UPHS. 25 patients with an ICD-9 code corresponding to a diagnosis of cystic fibrosis were excluded.

*Race/ethnicity*. Ethnicity was only captured as yes/no Hispanic. As a result, race and ethnicity were combined into a single variable. We limited our study to patients who were *non-Hispanic white* or *non-Hispanic black or African American*, as 87.8% of patients fell within these categories, with other categories having insufficient numbers of subjects to identify statistically meaningful relationships.

*BMI.* Body Mass Index (BMI) was calculated using an average of height and weight measurements, after eliminating values that fell outside 48 and 84 inches for height and 80 and 700 pounds for weight, or outside 5% of the patient’s median height measurement and 10% of the patient’s median weight measurement. BMI was classified into 5 categories: *not overweight or obese* (<25.0 kg/m^2^), *overweight* (25.0 to <30.0 kg/m^2^), *class 1 obese* (30.0 to <35.0 kg/m^2^), *class 2 obese* (35.0 to <40.0 kg/m^2^) and *class 3 obese* (>40.0 kg/m^2^).

*Health Insurance Type*. Health Insurance Type was defined as the most frequent codified billing method for each patient (ties were broken by recency), and was re-leveled into three groups: *Private Insurance*, *Medicare*, and *Medicaid*.

*Smoking status*. Smoking status was assigned into groups *Never*, *Quit*, *Passive*, *Yes* based on a report provided by PDS that is based on the full historical smoking data for each patient. Because only 81 patients were identified as having *Passive* exposure due to secondhand smoke, these patients were reclassified into the *Never* category.

**Comorbidity variable selection.**

To reduce the number of potential predictors, especially those that were collinear, we grouped comorbidity ICD-9 codes with the *icd* R package to map all non-procedural ICD-9 codes to Elixhauser Comorbidity Groups defined by the Agency for Healthcare Research and Quality (AHRQ) ^E1, E2^. We did not map codes to the *chronic pulmonary disease* category, as asthma falls under it. We did not map codes to the *obesity* category because we had a BMI variable. Due to IRB restrictions, we did not have ICD-9 codes related to the following five sensitive categories: *HIV*/*AIDS*, *alcohol*, *drug abuse*, *depression* and *psychosis*. After mapping ICD-9 codes to 23 AHRQ-Elixhauser categories, remaining ICD-9 codes were mapped to specific categories based on known or previously identified relationships with asthma: *allergic rhinitis, chronic bronchitis*, *emphysema*, *gastro-esophageal reflux disease (GERD)*, *obstructive sleep apnea*, *osteoporosis*, and *sinusitis* [Table E1] ^E3^.

Odds ratios for each of the 30 comorbidity categories were obtained using proportional odds logistic regression models to predict exacerbation count, while adjusting for demographic variables (age, race/ethnicity, sex, health insurance type, BMI, smoking history) [Table E3]. Eight comorbidity categories with Benjamini-Hochberg corrected p-values <10^-5^ were used in an adjusted model, which included all six demographic variables. All comorbidities remained significant except *congestive heart failure*, so it was not included in the final model.

**NHANES data and analysis.**  The *nhanesA* R package was used to obtain data from six 2-year cycles of NHANES (2001 to 2012) ^E4^. Subjects at least 20 years old and with asthma, based on affirmative responses to the questions “*Has a doctor or other health professional ever told you that you have asthma?*” and “*Do you still have asthma?*” were selected. Individuals were classified as having an asthma exacerbation based on an affirmative response to the question “*During the past 12 months, have you had to visit an emergency room or urgent care center because of asthma?*” Age, race/ethnicity, poverty-to-income ratio (PIR) (i.e. family income divided by poverty threshold), BMI, smoking status, comorbidities (i.e., affirmative response to having diseases asked in survey), and corticosteroid and respiratory agent prescription medications were captured using questionnaire and examination data. Race/ethnicity categories were those captured by NHANES: *non-Hispanic white, non-Hispanic black, Mexican American, other Hispanic*, or *other race*. PIR was classified into income *at or below poverty line* (≤1) or *above poverty line* (>1). BMI and smoking status were categorized as per the EHR-based data [Table 5]. The R *Survey* package was used to perform logistic regression while accounting for the study’s sampling design ^E5^. The final NHANES analysis was based on 2,071 individuals with complete data. Comorbidity categories with Benjamini-Hochberg corrected p-values below 0.05, as well as those selected for the EHR-based final model, were included in a final NHANES adjusted model.

**Figure E1**. Correlation among comorbidity and demographic factors is low (Spearman correlation < 0.36) suggesting their inclusion as independent variables in a multivariable model is appropriate.


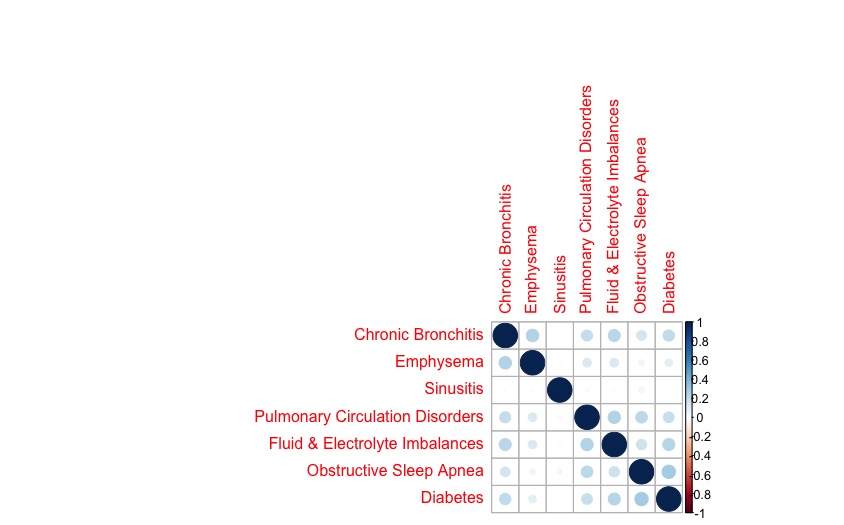
**
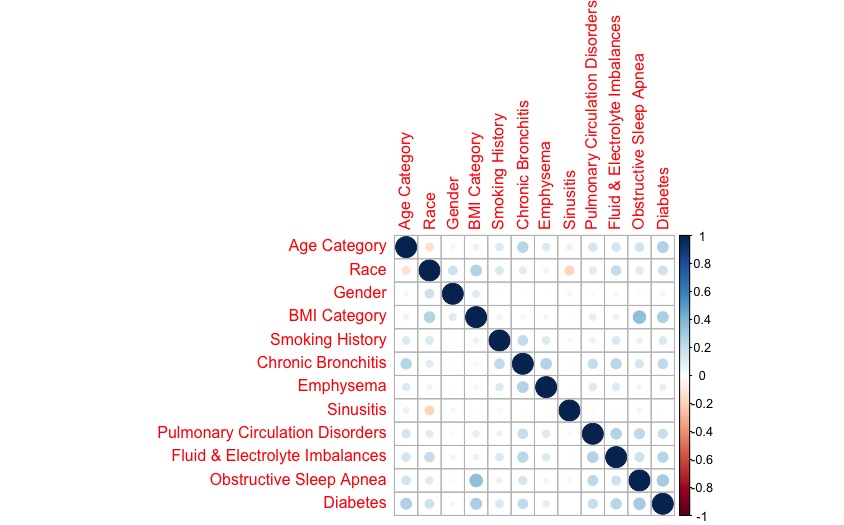
**

**Table E1**. ICD-9 codes assigned manually into comorbidity categories that have been previously associated with asthma.

| **Comorbidity** | **ICD-9 Codes** |
| --- | --- |
| Allergic rhinitis | 477.0, 477.1, 477.2, 477.8, 477.9 |
| Chronic bronchitis | 491.0, 491.1, 491.2, 491.21, 491.22, 491.8, 491.9 |
| Emphysema | 492.0, 492.8 |
| GERD | 530.11, 530.81 |
| Obstructive Sleep Apnea | 327.23 |
| Osteoporosis | 733.00, 733.01, 733.02, 733.03, 733.09, 733.7, 733.99 |
| Sinusitis | 461.0, 461.1, 461.2, 461.3, 461.8, 461.9, 473, 473.1, 473.2 473.3, 473.8, 473.9 |

**Table E2.** Patient Characteristics. Overall characteristics of complete cases used in analyses (i.e., Study Subjects) and those excluded due to missing BMI, health insurance type, and/or smoking history data (i.e., Incomplete Cases) corresponding to UPHS EHR data from 2011-2014. For each category, N (%) for raw data are shown.

|  |  | **Study Subjects**  **(Complete Cases)** | **Excluded Subjects**  **(Incomplete Cases)** | **Missing** |
| --- | --- | --- | --- | --- |
|  |  | N = 9,068 | N = 531 |  |
| **Exacerbation Count** |  |  |  | - |
|  | 0 | 6,042 (66.63) | 287 (54.05) |  |
|  | 1-2 | 2,639 (29.10) | 209 (39.36) |  |
|  | 3-4 | 273 (3.01) | 25 (4.71) |  |
|  | 5+ | 114 (1.26) | 10 (1.88) |  |
| **Age (years)** |  |  |  | - |
|  | 18-30 | 1,939 (21.38) | 155 (29.19) |  |
|  | 31-40 | 1,651 (18.21) | 97 (18.27) |  |
|  | 41-50 | 1,887 (20.81) | 122 (22.98) |  |
|  | 51-60 | 1,858 (20.49) | 89 (16.76) |  |
|  | 61-70 | 1,200 (13.23) | 51 (9.60) |  |
|  | 71-80 | 533 (5.88) | 17 (3.20) |  |
| **Race** |  |  |  | - |
|  | White | 4,397 (48.49) | 168 (31.64) |  |
|  | Black or African American | 4,671 (51.51) | 363 (68.36) |  |
| **Gender** |  |  |  | - |
|  | Male | 2,295 (25.31) | 187 (35.22) |  |
|  | Female | 6,773 (74.69) | 344 (64.78) |  |
| **BMI (**kg/m^2^) |  |  |  | 97 (1.01) |
|  | Not overweight or obese | 2,088 (23.03) | 122 (28.11) |  |
|  | Overweight (25 to < 30) | 2,487 (27.43) | 121 (27.88) |  |
|  | Class 1 obese (30 to < 35) | 1,962 (21.64) | 86 (19.82) |  |
|  | Class 2 obese (35 to < 40) | 1,203 (13.27) | 49 (11.29) |  |
|  | Class 3 obese (> 40) | 1,328 (14.64) | 56 (12.90) |  |
| **Health Insurance Type** |  |  |  | 45 (0.47) |
|  | Private insurance | 5,045 (55.64) | 199 (40.95) |  |
|  | Medicaid | 2,032 (22.41) | 208 (42.80) |  |
|  | Medicare | 1,991 (21.96) | 79 (16.26) |  |
| **Smoking History** |  |  |  | 417 (4.34) |
|  | Never | 5,019 (55.35) | 66 (57.89) |  |
|  | Quit | 2,714 (29.93) | 26 (22.81) |  |
|  | Yes | 1,335 (14.72) | 22 (19.30) |  |
| **Chronic Bronchitis** |  |  |  | - |
|  | No | 8,189 (90.31) | 458 (86.25) |  |
|  | Yes | 879 (9.69) | 73 (13.75) |  |
| **Emphysema** |  |  |  | - |
|  | No | 8,878 (97.90) | 523 (98.49) |  |
|  | Yes | 190 (2.10) | 8 (1.51) |  |
| **Sinusitis** |  |  |  | - |
|  | No | 5,832 (64.31) | 458 (86.25) |  |
|  | Yes | 3,236 (35.69) | 73 (13.75) |  |
| **Pulmonary Circulation Disorders** |  |  |  | - |
|  | No | 8,480 (93.52) | 510 (96.05) |  |
|  | Yes | 588 (6.48) | 21 (3.95) |  |
| **Fluid and Electrolyte Disorders** |  |  |  | - |
|  | No | 7,267 (80.14) | 428 (80.60) |  |
|  | Yes | 1,801 (19.86) | 103 (19.40) |  |
| **Obstructive Sleep Apnea** |  |  |  | - |
|  | No | 7,613 (83.95) | 490 (92.28) |  |
|  | Yes | 1,455 (16.05) | 41 (7.72) |  |
| **Diabetes (uncomplicated)** |  |  |  | ~~-~~ |
|  | No | 7,436 (82.00) | 455 (85.69) |  |
|  | Yes | 1,632 (18.00) | 76 (14.31) |  |

**Table E3.** Individual Comorbidity Adjusted Odds Ratios for Association with Asthma Exacerbations. Patients were assigned to having a comorbidity if at least one ICD-9 code corresponding to each category was indicated during an encounter. Number (N) of patients and corresponding percent (%) are shown, along with odds ratio for each comorbidity category’s association with asthma exacerbations while adjusting for *age, gender, race, BMI, health insurance type,* and *smoking history.*

| **Condition** | **N (%)** | **Adjusted Odds Ratio** | **P Value** |
| --- | --- | --- | --- |
| **Allergic Rhinitis** | 4,647 (51.25) | 1.07 (0.98, 1.17) | 1.3e-01 |
| **Chronic Bronchitis** | 879 (9.69) | 3.22 (2.78, 3.74)** | 7.2e-54 |
| **Emphysema** | 190 (2.1) | 2.40 (1.80, 3.19)** | 2.0e-09 |
| **GERD** | 3,717 (40.99) | 1.20 (1.09, 1.31)** | 1.0e-04 |
| **Obstructive Sleep Apnea** | 1,455 (16.05) | 1.43 (1.26, 1.62)** | 2.4e-08 |
| **Osteoporosis** | 590 (6.51) | 1.18 (0.99, 1.41) | 6.7e-02 |
| **Sinusitis** | 3,236 (35.69) | 1.53 (1.40, 1.68)** | 6.0e-20 |
| **Anemia** | 2,365 (26.08) | 1.07 (0.97, 1.19) | 1.9e-01 |
| **Blood loss anemia** | 517 (5.7) | 0.78 (0.63, 0.96)* | 2.1e-02 |
| **Congestive heart failure** | 740 (8.16) | 1.63 (1.39, 1.91)** | 1.7e-09 |
| **Coagulopathy** | 423 (4.66) | 1.14 (0.93, 1.39) | 2.0e-01 |
| **Diabetes (uncomplicated)** | 1,632 (18) | 1.52 (1.35, 1.72)** | 1.2e-11 |
| **Diabetes (complicated)** | 430 (4.74) | 1.30 (1.06, 1.59)* | 1.1e-02 |
| **Fluid and electrolyte disorders** | 1,801 (19.86) | 1.65 (1.47, 1.84)** | 2.3e-18 |
| **Hypertension (uncomplicated)** | 4,050 (44.66) | 1.28 (1.15, 1.42)** | 4.5e-06 |
| **Hypertension (complicated)** | 716 (7.9) | 1.41 (1.20, 1.66)** | 3.3e-05 |
| **Hypothyroidism** | 1,042 (11.49) | 0.94 (0.81, 1.08) | 4.0e-01 |
| **Liver failure** | 385 (4.25) | 1.09 (0.88, 1.35) | 4.3e-01 |
| **Lymphoma** | 81 (0.89) | 1.05 (0.67, 1.63) | 8.3e-01 |
| **Metastatic cancer** | 101 (1.11) | 1.09 (0.72, 1.63) | 6.8e-01 |
| **Other neurological disorders** | 840 (9.26) | 1.25 (1.08, 1.44)* | 2.4e-03 |
| **Paralysis** | 129 (1.42) | 0.95 (0.66, 1.37) | 7.8e-01 |
| **Pulmonary circulation disorders** | 588 (6.48) | 1.69 (1.42, 2.00)** | 1.9e-09 |
| **Peptic ulcer disease excluding bleeding** | 10 (0.11) | 2.02 (0.62, 6.37) | 2.4e-01 |
| **Peripheral vascular disorders** | 431 (4.75) | 1.05 (0.86, 1.29) | 6.4e-01 |
| **Renal failure** | 573 (6.32) | 1.40 (1.17, 1.66)** | 1.6e-04 |
| **Rheumatoid arthritis/collagen vascular diseases** | 530 (5.84) | 1.06 (0.88, 1.27) | 5.3e-01 |
| **Solid tumor without metastasis** | 732 (8.07) | 1.14 (0.97, 1.34) | 1.1e-01 |
| **Valvular disease** | 677 (7.47) | 1.11 (0.94, 1.31) | 2.2e-01 |
| **Weight loss** | 743 (8.19) | 1.20 (1.02, 1.41)* | 2.7e-02 |

*p < 0.05, ** p < 0.001

**Table E4**. Adjusted Parallel Slopes Test for Proportional Odds Logistic Regression used in EHR Analyses. Adjusted logistic regressions were performed to produce adjusted odds ratios (ORs) with exacerbation count > {1,3,5} as the binary outcome variable. Shown are adjusted ORs and 95% confidence intervals (CIs).

|  |  | **> 1 exacerbation** | **> 3 exacerbations** | **> 5 exacerbations** |
| --- | --- | --- | --- | --- |
|  | Controls | 6,042 (66.63) | 8,681 (95.73) | 8,954 (98.74) |
|  | Cases | 3,026 (33.37) | 387 (4.27) | 114 (1.26) |
| **Age (years)** |  |  |  |  |
|  | 10 years^†^ | 1.15 (1.11, 1.19)** | 1.06 (0.97, 1.16) | 0.86 (0.73, 1.01) |
| **Race** |  |  |  |  |
|  | White | Reference | Reference | Reference |
|  | Black or African American | 1.13 (1.01, 1.26)* | 1.71 (1.31, 2.24)** | 4.44 (2.38, 8.96)** |
| **Gender** |  |  |  |  |
|  | Male | Reference | Reference | Reference |
|  | Female | 1.08 (0.97, 1.20) | 0.85 (0.66, 1.10) | 0.69 (0.44, 1.11) |
| **BMI (**kg/m^2^) |  |  |  |  |
|  | Not overweight or obese | Reference | Reference | Reference |
|  | Overweight (25 to < 30) | 1.05 (0.92, 1.20) | 0.96 (0.69, 1.33) | 0.92 (0.48, 1.79) |
|  | Class 1 obese (30 to < 35) | 1.11 (0.97, 1.28) | 1.02 (0.73, 1.43) | 1.07 (0.57, 2.06) |
|  | Class 2 obese (35 to < 40) | 1.10 (0.93, 1.30) | 1.17 (0.81, 1.69) | 1.03 (0.51, 2.11) |
|  | Class 3 obese (> 40) | 1.39 (1.18, 1.64)** | 0.80 (0.54, 1.19) | 0.86 (0.42, 1.80) |
| **Health Insurance Type** |  |  |  |  |
|  | Private insurance | Reference | Reference | Reference |
|  | Medicaid | 0.91 (0.80, 1.04) | 1.72 (1.29, 2.30)** | 2.47 (1.43, 4.40)* |
|  | Medicare | 0.83 (0.72, 0.95)* | 0.96 (0.70, 1.31) | 2.26 (1.25, 4.19)* |
| **Smoking History** |  |  |  |  |
|  | Never | Reference | Reference | Reference |
|  | Quit | 0.98 (0.88, 1.09) | 1.11 (0.87, 1.43) | 0.89 (0.55, 1.41) |
|  | Yes | 1.20 (1.04, 1.38)* | 0.81 (0.58, 1.11) | 0.71 (0.40, 1.22) |
| **Chronic Bronchitis** |  |  |  |  |
|  | No | Reference | Reference | Reference |
|  | Yes | 2.60 (2.22, 3.06)** | 2.78 (2.10, 3.65)** | 2.58 (1.58, 4.18)** |
| **Emphysema** |  |  |  |  |
|  | No | Reference | Reference | Reference |
|  | Yes | 1.40 (1.02, 1.93)* | 1.33 (0.82, 2.10) | 1.45 (0.65, 2.97) |
| **Sinusitis** |  |  |  |  |
|  | No | Reference | Reference | Reference |
|  | Yes | 1.48 (1.34, 1.63)** | 1.65 (1.33, 2.06)** | 2.07 (1.39, 3.06)** |
| **Pulmonary Circulation Disorders** |  |  |  |  |
|  | No | Reference | Reference | Reference |
|  | Yes | 1.21 (1.00, 1.46) | 1.18 (0.84, 1.64) | 1.31 (0.77, 2.19) |
| **Fluid and Electrolyte Disorders** |  |  |  |  |
|  | No | Reference | Reference | Reference |
|  | Yes | 1.32 (1.17, 1.49)** | 1.61 (1.26, 2.04)** | 1.88 (1.23, 2.89)* |
| **Obstructive Sleep Apnea** |  |  |  |  |
|  | No | Reference | Reference | Reference |
|  | Yes | 1.12 (0.98, 1.29) | 1.28 (0.96, 1.68) | 1.52 (0.94, 2.46) |
| **Diabetes (uncomplicated)** |  |  |  |  |
|  | No | Reference | Reference | Reference |
|  | Yes | 1.24 (1.10, 1.41)** | 1.45 (1.12, 1.87)* | 1.92 (1.22, 3.03)* |

*p < 0.05, ** p < 0.001

^†^ Odds ratios shown are for a 10-year increase in age

**Table E5**. ICD-9 codes considered in broader *chronic airway obstruction exacerbation* definition and number of times assigned at an encounter that also contained an oral steroid prescription.

| ICD-9 Code | Description | # of times as primary code at same visit as prescription for oral steroid |
| --- | --- | --- |
| 493.9 | Asthma, unspecified type | 2,874 |
| 493.92 | Asthma, unspecified type, with (acute) exacerbation | 2,084 |
| 491.21 | Obstructive chronic bronchitis with (acute) exacerbation | 704 |
| 466 | Acute bronchitis | 493 |
| 496 | Chronic airway obstruction, not elsewhere classified | 345 |
| 493.22 | Chronic obstructive asthma with (acute) exacerbation | 301 |
| 786.05 | Shortness of breath | 197 |
| 493.1 | Intrinsic asthma, unspecified | 92 |
| 493.82 | Cough variant asthma | 72 |
| 490 | Bronchitis, not specified as acute or chronic | 70 |
| 493.2 | Chronic obstructive asthma, unspecified | 52 |
| 493.91 | Asthma, unspecified type, with status asthmaticus | 51 |
| 493 | Extrinsic asthma, unspecified | 49 |
| 786.07 | Wheezing | 49 |
| 518.81 | Acute respiratory failure | 43 |
| 493.02 | Extrinsic asthma with (acute) exacerbation | 35 |
| 492.8 | Other emphysema | 30 |
| 493.11 | Intrinsic asthma with status asthmaticus | 24 |
| 493.12 | Intrinsic asthma with (acute) exacerbation | 24 |
| 518.84 | Acute and chronic respiratory failure | 18 |
| 493.01 | Extrinsic asthma with status asthmaticus | 17 |
| 491.22 | Obstructive chronic bronchitis with acute bronchitis | 16 |
| 491.9 | Unspecified chronic bronchitis | 9 |
| 519.11 | Acute bronchospasm | 9 |
| 491 | Simple chronic bronchitis | 7 |
| 491.2 | Obstructive chronic bronchitis without exacerbation | 7 |
| 493.81 | Exercise induced bronchospasm | 6 |
| 491.8 | Other chronic bronchitis | 3 |
| 493.21 | Chronic obstructive asthma with status asthmaticus | 3 |

**Table E6**. UPHS EHR Patient Characteristics by *Chronic Airway Obstruction Exacerbation* Count. For each category, N (%) for raw data are shown.

|  |  | **0** | **1-2** | **3-4** | **5+** |
| --- | --- | --- | --- | --- | --- |
|  |  | N = 5,443 | N = 2,824 | N = 473 | N = 328 |
| **Age (years)** |  |  |  |  |  |
|  | 18-30 | 1,462 (26.86) | 421 (14.91) | 34 (7.19) | 22 (6.71) |
|  | 31-40 | 1,055 (19.38) | 501 (17.74) | 60 (12.68) | 35 (10.67) |
|  | 41-50 | 1,095 (20.12) | 581 (20.57) | 125 (26.43) | 86 (26.22) |
|  | 51-60 | 922 (16.94) | 708 (25.07) | 134 (28.33) | 94 (28.66) |
|  | 61-70 | 641 (11.78) | 411 (14.55) | 81 (17.12) | 67 (20.43) |
|  | 71-80 | 268 (4.92) | 202 (7.15) | 39 (8.25) | 24 (7.32) |
| **Race** |  |  |  |  |  |
|  | White | 2,693 (49.48) | 1,399 (49.54) | 231 (48.84) | 74 (22.56) |
|  | Black or African American | 2,750 (50.52) | 1,425 (50.46) | 242 (51.16) | 254 (77.44) |
| **Gender** |  |  |  |  |  |
|  | Male | 1,423 (26.14) | 671 (23.76) | 111 (23.47) | 90 (27.44) |
|  | Female | 4,020 (73.86) | 2,153 (76.24) | 362 (76.53) | 238 (72.56) |
| **BMI (kg/m^2^)** |  |  |  |  |  |
|  | Not overweight or obese | 1,364 (25.06) | 563 (19.94) | 80 (16.91) | 81 (24.70) |
|  | Overweight (25 to < 30) | 1,551 (28.50) | 737 (26.10) | 121 (25.58) | 78 (23.78) |
|  | Class 1 obese (30 to < 35) | 1,149 (21.11) | 626 (22.17) | 118 (24.95) | 69 (21.04) |
|  | Class 2 obese (35 to < 40) | 684 (12.57) | 394 (13.95) | 77 (16.28) | 48 (14.63) |
|  | Class 3 obese (> 40) | 695 (12.77) | 504 (17.85) | 77 (16.28) | 52 (15.85) |
| **Health Insurance Type** |  |  |  |  |  |
|  | Private insurance | 3,169 (58.22) | 1,561 (55.28) | 222 (46.93) | 93 (28.35) |
|  | Medicaid | 1,241 (22.80) | 569 (20.15) | 110 (23.26) | 112 (34.15) |
|  | Medicare | 1,033 (18.98) | 694 (24.58) | 141 (29.81) | 123 (37.50) |
| **Smoking History** |  |  |  |  |  |
|  | Never | 3,215 (59.07) | 1,491 (52.80) | 207 (43.76) | 106 (32.32) |
|  | Quit | 1,483 (27.25) | 884 (31.30) | 180 (38.05) | 167 (50.91) |
|  | Yes | 745 (13.69) | 449 (15.90) | 86 (18.18) | 55 (16.77) |
| **Chronic Bronchitis** |  |  |  |  |  |
|  | No | 5,240 (96.27) | 2,459 (87.08) | 333 (70.40) | 157 (47.87) |
|  | Yes | 203 (3.73) | 365 (12.92) | 140 (29.60) | 171 (52.13) |
| **Emphysema** |  |  |  |  |  |
|  | No | 5,386 (98.95) | 2,760 (97.73) | 450 (95.14) | 282 (85.98) |
|  | Yes | 57 (1.05) | 64 (2.27) | 23 (4.86) | 46 (14.02) |
| **Sinusitis** |  |  |  |  |  |
|  | No | 3,743 (68.77) | 1,652 (58.50) | 253 (53.49) | 184 (56.10) |
|  | Yes | 1,700 (31.23) | 1,172 (41.50) | 220 (46.51) | 144 (43.90) |
| **Pulmonary Circulation Disorders** |  |  |  |  |  |
|  | No | 5,199 (95.52) | 2,615 (92.60) | 413 (87.32) | 253 (77.13) |
|  | Yes | 244 (4.48) | 209 (7.40) | 60 (12.68) | 75 (22.87) |
| **Fluid and Electrolyte Disorders** |  |  |  |  |  |
|  | No | 4,605 (84.60) | 2,171 (76.88) | 325 (68.71) | 166 (50.61) |
|  | Yes | 838 (15.40) | 653 (23.12) | 148 (31.29) | 162 (49.39) |
| **Obstructive Sleep Apnea** |  |  |  |  |  |
|  | No | 4,744 (87.16) | 2,290 (81.09) | 357 (75.48) | 222 (67.68) |
|  | Yes | 699 (12.84) | 534 (18.91) | 116 (24.52) | 106 (32.32) |
| **Diabetes (uncomplicated)** |  |  |  |  |  |
|  | No | 4,698 (86.31) | 2,210 (78.26) | 342 (72.30) | 186 (56.71) |
|  | Yes | 745 (13.69) | 614 (21.74) | 131 (27.70) | 142 (43.29) |

**Table E7**. Factors Associated with *Chronic Airway Obstruction Exacerbation* Count. Proportional odds logistic regressions were used in unadjusted and adjusted models to obtain crude and adjusted odds ratios (ORs) with airway obstruction exacerbation count as the outcome (outcome levels: 0, 1-2, 3-4, 5+ exacerbations). Shown are ORs, 95% confidence intervals (CIs) and p-values for the adjusted model.

|  |  | **Crude Odds Ratio** | **Adjusted Odds Ratio** | **P Value for Adjusted OR** |
| --- | --- | --- | --- | --- |
| **Age** |  |  |  |  |
|  | 10 years^†^ | 1.21 (1.18, 1.25)** | 1.16 (1.12, 1.20)** | 3.4e-17 |
| **Race** |  |  |  |  |
|  | White | Reference | Reference |  |
|  | Black or African American | 1.16 (1.07, 1.26)** | 1.04 (0.94, 1.16) | 4.6e-01 |
| **Gender** |  |  |  |  |
|  | Male | Reference | Reference |  |
|  | Female | 1.1 (1, 1.21)* | 1.06 (0.96, 1.17) | 2.5e-01 |
| **BMI (kg/m^2^)** |  |  |  |  |
|  | Not overweight or obese | Reference | Reference |  |
|  | Overweight (25 to < 30) | 1.12 (1, 1.27) | 1.08 (0.95, 1.22) | 2.3e-01 |
|  | Class 1 obese (30 to < 35) | 1.32 (1.17, 1.5)** | 1.14 (1.00, 1.30) | 5.0e-02 |
|  | Class 2 obese (35 to < 40) | 1.42 (1.24, 1.64)** | 1.17 (1.01, 1.37)* | 4.4e-02 |
|  | Class 3 obese (> 40) | 1.64 (1.43, 1.88)** | 1.33 (1.14, 1.55)** | 2.7e-04 |
| **Health Insurance Type** |  |  |  |  |
|  | Private insurance | Reference | Reference |  |
|  | Medicaid | 1.16 (1.04, 1.28)* | 0.95 (0.84, 1.08) | 4.2e-01 |
|  | Medicare | 1.66 (1.5, 1.83)** | 0.80 (0.70, 0.91)** | 8.6e-04 |
| **Smoking History** |  |  |  |  |
|  | Never | Reference | Reference |  |
|  | Quit | 1.57 (1.43, 1.72)** | 1.09 (0.98, 1.20) | 9.5e-02 |
|  | Yes | 1.45 (1.28, 1.63)** | 1.13 (0.99, 1.29) | 7.0e-02 |
| **Chronic Bronchitis** |  |  |  |  |
|  | No | Reference | Reference |  |
|  | Yes | 7.44 (6.47, 8.56)** | 4.74 (4.07, 5.53)** | 4.2e-88 |
| **Emphysema** |  |  |  |  |
|  | No | Reference | Reference |  |
|  | Yes | 5.31 (4, 7.05)** | 1.77 (1.32, 2.38)** | 1.5e-04 |
| **Sinusitis** |  |  |  |  |
|  | No | Reference | Reference |  |
|  | Yes | 1.61 (1.48, 1.75)** | 1.61 (1.47, 1.76)** | 3.5e-25 |
| **Pulmonary Circulation Disorders** |  |  |  |  |
|  | No | Reference | Reference |  |
|  | Yes | 2.63 (2.23, 3.09)** | 1.35 (1.14, 1.61)** | 6.5e-04 |
| **Fluid and Electrolyte Disorders** |  |  |  |  |
|  | No | Reference | Reference |  |
|  | Yes | 2.17 (1.96, 2.4)** | 1.42 (1.27, 1.59)** | 9.5e-10 |
| **Obstructive Sleep Apnea** |  |  |  |  |
|  | No | Reference | Reference |  |
|  | Yes | 1.87 (1.68, 2.09)** | 1.09 (0.96, 1.24) | 1.9e-01 |
| **Diabetes (uncomplicated)** |  |  |  |  |
|  | No | Reference | Reference |  |
|  | Yes | 2.18 (1.96, 2.42)** | 1.26 (1.12, 1.42)** | 1.3e-04 |

*p < 0.05, ** p < 0.001

^†^ Odds ratios shown are for a 10-year increase in age

**Table E8**. NHANES Patient Characteristics. For each category, N (weighted %) are shown.

|  |  | **No Exacerbation** | **Exacerbation** |
| --- | --- | --- | --- |
|  |  | N=1,753 | N=318 |
| **Age (years)** |  |  |  |
|  | 20-30 | 378 (22.35) | 74 (25.13) |
|  | 31-40 | 279 (17.39) | 64 (22.27) |
|  | 41-50 | 272 (19.86) | 54 (19.64) |
|  | 51-60 | 294 (19.02) | 58 (15.99) |
|  | 61-70 | 267 (11.77) | 41 (10.64) |
|  | 71+ | 263 (9.62) | 27 (6.32) |
| **Race** |  |  |  |
|  | Non-Hispanic White | 982 (76.15) | 123 (57.17) |
|  | Non-Hispanic Black | 418 (11.97) | 107 (21.98) |
|  | Hispanic | 112 (3.58) | 38 (9.79) |
|  | Mexican American | 146 (3.30) | 33 (6.23) |
|  | Other | 95 (5.00) | 17 (4.82) |
| **Gender** |  |  |  |
|  | Male | 657 (37.60) | 96 (26.91) |
|  | Female | 1096 (62.40) | 222 (73.09) |
| **BMI (kg/m^2^)** |  |  |  |
|  | Not overweight or obese | 424 (27.25) | 61 (20.65) |
|  | Overweight (25 to < 30) | 404 (22.28) | 73 (23.76) |
|  | Class 1 obese (30 to < 35) | 214 (11.53) | 41 (12.40) |
|  | Class 2 obese (35 to < 40) | 219 (10.71) | 64 (19.24) |
|  | Class 3 obese (> 40) | 492 (28.24) | 79 (23.95) |
| **Poverty Income Ratio** |  |  |  |
|  | ≤1 | 465 (19.36) | 126 (31.94) |
|  | >1 | 1288 (80.64) | 192 (68.06) |
| **Smoking History** |  |  |  |
|  | Never | 847 (49.67) | 132 (43.88) |
|  | Quit | 474 (25.86) | 84 (25.88) |
|  | Yes | 432 (24.46) | 102 (30.24) |
| **Chronic Bronchitis** |  |  |  |
|  | No | 1338 (76.42) | 188 (58.78) |
|  | Yes | 415 (23.58) | 130 (41.22) |
| **Emphysema** |  |  |  |
|  | No | 1589 (92.35) | 277 (89.61) |
|  | Yes | 164 (7.65) | 41 (10.39) |
| **Anemia** |  |  |  |
|  | No | 1667 (96.16) | 276 (87.76) |
|  | Yes | 86 (3.84) | 42 (12.24) |
| **Diabetes** |  |  |  |
|  | No | 1478 (88.72) | 254 (85.09) |
|  | Yes | 275 (11.28) | 64 (14.91) |

**Table E9**. NHANES Comorbidity Adjusted Odds Ratios. N (%) and odds ratio for each comorbidity category after adjusting for *age, gender, race, BMI, financial class,* and *smoking history* are shown*.*

| **Condition** | **Patients with Condition**  **N (%)** | **Adjusted Odds Ratio** | **P Value** |
| --- | --- | --- | --- |
| **Anemia** | 128 (4.91) | 2.93 (1.70, 5.05)** | 1.1e-04 |
| **Trouble seeing** | 576 (24.48) | 1.20 (0.89, 1.62) | 2.3e-01 |
| **Arthritis** | 850 (36.41) | 1.75 (1.17, 2.63)* | 6.8e-03 |
| **Congestive Heart Failure** | 135 (5.02) | 1.97 (1.11, 3.48)* | 2.0e-02 |
| **Coronary Heart Disease** | 116 (4.82) | 1.33 (0.71, 2.50) | 3.7e-01 |
| **Angina** | 131 (5.12) | 1.91 (1.18, 3.10)* | 8.6e-03 |
| **Heart Attack** | 153 (5.58) | 1.62 (0.92, 2.85) | 9.4e-02 |
| **Stroke** | 113 (4.62) | 1.05 (0.63, 1.74) | 8.5e-01 |
| **Emphysema** | 205 (8) | 1.90 (1.24, 2.91)* | 3.2e-03 |
| **Chronic Bronchitis** | 545 (25.82) | 2.56 (1.79, 3.66)** | 2.6e-07 |
| **Liver Condition** | 96 (4.1) | 2.09 (1.04, 4.22)* | 3.9e-02 |
| **Cancer** | 254 (12.6) | 1.74 (1.07, 2.84)* | 2.6e-02 |
| **Hypertension** | 934 (38.49) | 1.08 (0.71, 1.62) | 7.1e-01 |
| **Diabetes** | 339 (11.74) | 1.17 (0.78, 1.75) | 4.5e-01 |

*p < 0.05, ** p < 0.001

**Table E10**. Medications NHANES Subjects Reported Taking in Past Month. For each category, N (%) for raw data are shown.

| **Medication Class** | **No Exacerbation** | **Exacerbation** |
| --- | --- | --- |
| Anticholinergic | 75 (3.30) | 24 (6.50) |
| Anti-IgE | 0 (0.00) | 0 (0.00) |
| Cromolyn | 4 (0.29) | 2 (0.78) |
| Epinephrine | 0 (0.00) | 1 (0.25) |
| ICS | 146 (7.83) | 37 (13.86) |
| ICS/LABA | 241 (14.52) | 71 (21.93) |
| LABA | 49 (2.87) | 11 (4.40) |
| LTRA | 159 (9.43) | 48 (15.09) |
| OCS | 50 (2.13) | 33 (10.30) |
| PDE-4 inhibitor | 0 (0.00) | 0 (0.00) |
| SABA | 511 (28.15) | 157 (47.06) |
| SABA/anticholinergic | 53 (2.64) | 19 (5.97) |
| Terbutaline | 1 (0.03) | 1 (0.23) |
| Xanthine | 33 (1.46) | 10 (2.56) |

References

E1. Wasey, J.O. *icd: Tools for Working with ICD-9 and ICD-10 Codes, and Finding Comorbidities*. R package version 3.2.0. 2018; Available from: <https://CRAN.R-project.org/package=icd>.

E2. *HCUP Elixhauser Comorbidity Software. Healthcare Cost and Utilization Project (HCUP).* Agency for Healthcare Research and Quality 2017; Available from: <http://www.hcup-us.ahrq.gov/toolssoftware/comorbidity/comorbidity.jsp>.

E3. Denlinger, L.C., B.R. Phillips, S. Ramratnam, K. Ross, N.R. Bhakta, J.C. Cardet, M. Castro, et al., *Inflammatory and Comorbid Features of Patients with Severe Asthma and Frequent Exacerbations.* Am J Respir Crit Care Med, 2017. **195**(3): p. 302-313.

E4. Endres, C.J. *nhanesA: NHANES Data Retrieval.* R package version 0.6.4.4. 2018; Available from: <https://CRAN.R-project.org/package=nhanesA>.

E5. Lumley, T. *survey: analysis of complex survey samples*. R package version 3.32 2017; Available from: <https://CRAN.R-project.org/package=survey>.
